# Dazomet application suppressed watermelon wilt by the altered soil microbial community

**DOI:** 10.1038/s41598-020-78839-5

**Published:** 2020-12-10

**Authors:** Feiying Zhu, Jiling Xiao, Yi Zhang, Lin Wei, Zhihuai Liang

**Affiliations:** 1 Hunan Agricultural Biotechnology Research Institute, Hunan Academy of Agricultural Sciences, Changsha, 410125 People’s Republic of China; 2 Institute of Plant Protection, Hunan Academy of Agricultural Sciences, Changsha, Hunan Province 410125 People’s Republic of China

**Keywords:** Plant sciences, Plant stress responses, Abiotic, Agroecology

## Abstract

*Fusarium* wilt disease causes severe decline of watermelon yield and quality. Researches have been reported that soil fumigation with dazomet can help control crop disease. Firstly, we discovered that the dazomet application suppressed watermelon wilt in field experiment compared to the control group. While the importance of microbial community in regulating plant health has been rising up, we therefore focused on examining the soil microbial diversity at six different sampling times after dazomet application by using Illumina MiSeq platform. Remarkably, our research results showed that some beneficial microbial genera have been altered, and these beneficial microbial genera have dominated the entire community, such as *Nitrolancea*, *Pseudomonas and Penicillium* after dazomet application. Instead, the relative abundance of *Fusarium* genus and the pathogen FON (*Fusarium oxysporum f. sp. niveum*, FON) had the decreased. As there was a significant accumulation of AP (available soil phosphorus) after dazomet application, we noticed that the beneficial microbes as *Bacillus*, *Nitrolancea*, *Paenibacillus* and *Penicillium* have significant positive correlation with AP but negatively related to morbidity. Together, these results demonstrate that the altered soil microbial community structure by dazomet application is critical to suppress watermelon *Fusarium* wilt. Thus, our results will drive investigations aimed to deploy interaction of microbiota contribute and plant immunity.

## Introduction

Watermelon (*Citrullus lanatus*) plants are sensitive to *Fusarium* wilt disease caused by *Fusarium oxysporum f. sp. Niveum* (FON), which poses a serious threat to decline watermelon yield and quality^1^. The symptoms of *Fusarium* wilt diseased plants with rotted, discolored root and the vascular bundle became brown^2^. Different techniques, such as grafting onto disease-resistant rootstocks, chemical control^3^, biological control and use of disease-resistant cultivars^4^ are utilized to overcome this kind of disease. Dazomet is considered as one of a comprehensive soil fumigation disinfectant to efficiently control fungal disease, pests and weeds^5^. For example, laboratory studies and field trials reported by Mao^6^ have indicated that soil fumigation with dazomet could be applied in integrated pest management for controlling ginger bacterial wilt in China. Nicola^7^ confirmed that fumigation with dazomet modifies the composition of beneficial microorganisms in the soil of apple orchards affected by replant disease. In addition, Huang^8^ demonstrated that reductive soil disinfestation-related treatments improved the soil metabolic activity and functional diversity.

Recently, researches have focused on the mutual and the recognition between host plants and pathogens, the recognition competition between host plant defense factors and pathogenic factors. Relative studies have shown that the plant immune system shapes the microbiome, and these microbiomes can increase the plant immune capacity^9^. Furthermore, several studies represented that regulating the ecological balance of soil microorganisms can be conducive to inhibiting crop diseases^10^. However, the basis for how the soil microbes changed in the case of the watermelon defense against disease has remained uncharacterized. Based on our previous experiments of different fertilizer combinations on occurrence of watermelon *Fusarium* wilt and suggested that soil microbial community structure have played an important role in plant growth^11,12^. We hypothesis that whether the soil environmental factors or the microbial community constructers altered that effect plant immune system to defense Fusarium wilt after dazomet application in continues watermelon cropping soil. Therefore, in this experiment, we focused on the soil microbial diversity at 6 different sampling times after dazomet application by using Illumina MiSeq platform to explore the dynamic changes of soil microbial community and identify its importance to suppress watermelon *Fusarium* wilt.

## Materials and methods

### Experimental design

This study was conducted at Gaoqiao Scientific Research Base of the Hunan Academy of Agricultural Sciences in the city of Changsha (112°58′42ʺ E, 28°11′49ʺ N), Hunan Province in China in 2018 and 2019. The soil was sandy loam. The trial crop was watermelon cultivars zaojia 8424, which was provided by Xinjiang Farmer Seed Technology Co., Ltd. China. The dazomet was provided by Beijing Sino Green Agri-Biotech Co., Ltd. Six greenhouses (30 m × 6 m) with the same background, which were cultivated watermelon under monocropping system for five years, were selected. Three of them were treated with dazomet as three replicates, others were as control group. The routine cultivation managements in all the greenhouses were the same. Every March before transplanting the watermelon seedlings, 6 kg (98% C_5_H_18_N_2_S_2_) of dazomet were applied to one greenhouse, which was then tilled the soil by a rotary immediately after spraying. Controlling the depth of tillage soil 0–20 cm to ensure that dazomet was evenly mixed into the tillage layer. As soon as the soil temperature is above 8 °C, film mulching was set up to maintain the fumes of dazomet into the soil to kill most of the soil organisms, as well as to maintain the soil moisture content at approximately 40% for the germination and growth of weeds and pathogens. After 20 days, the film was uncovered and the greenhouse was kept ventilated. Then 15 days later, the watermelon seedlings nutrition bowl was cultivated and transplanted into the greenhouse. We planted the watermelon in the greenhouse with 50–60 cm plant spacing to enable pruning the climbing vines.

We designed six different sampling times as following: 1 (March 6th, 2018, before dazomet treatment), 2 (April 24th, 2018, watermelon seedling stage), 3 (May 3rd, 2018, *Fusarium* wilt symptom appearance), 4 (March 6th, 2019, before dazomet treatment), 5 (April 22th, 2019, watermelon seedling stage), 6 (April 29th, 2019, *Fusarium* wilt symptom appearance). For each replicate, nine independent soil samples within depth of 0–20 cm in the shape of “S” from each greenhouse were pooled. Three greenhouses within same treatment regarded as three independent replicates. DAZ represents dazomet treatment group and CK represents the control group without dazomet application but using same conventional planting system. All the soil samples from greenhouses were packed into sealed sterile bags separately and brought back to the laboratory. After removing the plant roots and stones from the samples, we sieved them with a 20-μm mesh, and then divided each sample into three parts. Two of them were placed in sterile centrifuge tubes, stored at − 80 °C for sequencing analysis and Q-PCR test. While the other was used for measuring the soil properties, stored at room temperature. We have collected total of 36 samples in six different sampling times.

### Field disease investigation

The incidence of Fusarium wilt was calculated during the whole watermelon onset period (Started from plants with rotted, discolored root and the vascular bundle became brown until the whole plant died). The disease incidence (%) = (number of infected plants/total number of surveys) × 100%.

### Determination of soil physical and chemical properties

The soil characteristics are listed in Supplementary Table S1. Soil pH was determined in a soil: water ratio of 1:2.5 (wt./vol) using a pH meter (BPH-220, Bell Instrument Equipment Co. Ltd., Dalian, LN, China). To extract the water-soluble salts from the soil, samples of 1 mm sieved and air-dried soil weighing 20.00 g were placed in a 250 ml Erlenmeyer flask, 100 ml of distilled water was added (water: soil ratio of 5:1). Then put it into a dry triangular bottle after shaking for 5 min which was used for the determination of salt. A total of 30 ml of the soil leachate was placed in 50 ml of burnout solution. The solution temperature was measured, and then the conductivity of the solution was determined using a conductometer. The soil organic matter (SOM) was determined by oxidation with potassium dichromate by DF-101S heat collecting constant temperature magnetic stirrer (Gongyi yuhua instrument Co., Ltd, Gongyi, HN, China). Total P and K and available P and K concentrations in the soil were determined by ICP-AES (PerkinElmer 2100DV, PerkinElmer, Waltham, MA, USA) after the soils were digested using concentrated HNO_3_-HF-HClO_4_. Total nitrogen (N) and available nitrogen (AN) in the soil were determined by the Kjeldahl method and the alkali diffusion method, respectively (China Agricultural Technology Extension Service Center, 2014).

### Soil microbial diversity analysis

Total genomic DNA was extracted from the soil samples using the E.Z.N.A Soil DNA kit (Omega Bio-tech, Norcross, GA, USA) according to manufacturer’s protocols. The final DNA concentration and purity were determined using a Nanodrop 2000 UV–Vis spectrophotometer (Thermo Scientific, Wilmington, DE, USA), and the DNA quality was checked by 1% agarose gel electrophoresis. Distinct regions of the 16S rRNA gene (V3-V4) and ITS1 were amplified by PCR (ABI Geneamp 9700, Applied Biosystems, Inc., Carlsbad, CA, USA) using specific primers (16S: 338F (5′-ACTCCTACGGGAGGCAGCAG-3′), 806R (5′-GGACTACHVGGGTWTCTAAT-3′); ITS1F (5′-CTTGGTCATTTAGAGGAAGTAA-3′), ITS2R (5′-GCTGCGTTCTTCATCGATGC-3′)), separately. The PCRs were conducted using the following programme: 3 min of denaturation at 95 °C, 27 cycles of 30 s at 95 °C for ITS1 rRNA gene/35 cycles of 30 s at 95 °C for 16S rRNA gene, 30 s of annealing at 55 °C, and 45 s of elongation at 72 °C with a final extension at 72 °C for 10 min, 10 °C ∞. PCR products were extracted from a 2% agarose gel and further purified using the AxyPrep DNA Gel Extraction Kit (Axygen Biosciences, Union City, CA, USA), followed by quantification using the QuantiFluor-ST kit (Promega, Madison, MI, USA) according to the manufacturer’s protocol.

Purified amplicons were pooled in equimolar amounts and sequenced (paired-end; 2 × 300 bp) on an Illumina MiSeq platform (Illumina, San Diego, CA, USA) according to the standard protocols of the Majorbio Bio-Pharm Technology Co. Ltd. (Shanghai, China). The raw reads were deposited into the NCBI Sequence Read Archive (SRA) database (Accession Number: SRP268536).

### Quantitative detection of FON by real-time PCR

Distinct regions of the FON rRNA genes were amplified by PCR (Bio-Rad T100 Thermal Cycler, Bio-Rad Laboratories, Inc. Hercules, CA, USA) using specific primers (Fonq-F(5′- GTTGCTTACGGTTCTAACTGTGC -3′), Fonp1-R(5′- CTGGTACGGAATGGCCGATCAG -3′)) . Then the PCR products were used as templates to construct the standard curve of the fluorescence quantitative PCR (Bio-Rad iQ5 Optical Module, Bio-Rad Laboratories, Inc. Hercules, CA, USA) using primers (Fonq-F(5′- GTTGCTTACGGTTCTAACTGTGC -3′), Fonq-R(5′- GGTACTTGGAAGGAATTGTGGG -3′)). A 1446 bp DNA fragments containing the qPCR target sequence was amplified from soil DNA by conventional PCR (initial incubation at 94 °C for 4 min, followed by 18 cycles of 94 °C 40 s, 60 °C 40 s, 72 °C 70 s, and a final extension at 72 °C for 10 min). The PCR products were used as templates to construct the standard curve of the fluorescence quantitative PCR (reaction consisted of an initial incubation at 95 °C for 1 min, followed by 40 cycles of 95 °C 15 s, 60 °C 30 s, 72 °C 30 s). The fluorescence intensity was monitored every 0.5 °C between 65 °C-95°C to making standard melting curve^13^.

### Data analysis

Raw FASTQ files were demultiplexed, quality-filtered by Trimmomatic and merged by FLASH with the following criteria: (i) The reads were truncated at any site receiving an average quality score < 20 over a 50 bp sliding window, and the truncated reads shorter than 50 bp were discarded; (ii) exact barcode matching, primers were exactly matched, and reads containing ambiguous bases were removed; (iii) sequences with over 10 bp of overlap were merged according to their overlap sequence. The singletons were removed for further analyses. Operational taxonomic units (OTUs) were clustered with a 97% similarity cut-off using UPARSE Version 7.1 (http://drive5.com/uparse/) and chimeric sequences were identified and removed using UCHIME ^14^. The taxonomy of each 16S rRNA gene sequence was analyzed by the RDP Classifier algorithm against the Silva (SSU123) 16S rRNA database using a confidence threshold of 70%. The taxonomy of each ITS region sequence was analyzed by the RDP Classifier algorithm against the UNITE (8.0) ITS rRNA database using a confidence threshold of 70%.

The diversity analysis for the sequencing data were performed on the free online platform of Majorbio Cloud Platform (www.majorbio.com) based using the Qiime2 software^15^ (https://qiime2.org/). For example, the sobs index of rarefaction curve, alpha diversity (student’s t-test shannon index) and beta diversity of (NMDS, non-metric multidimensional scaling analysis) with ANOSIM statistical analysis to compare the composition between treatments on OTU level. Moreover, the significant difference of microbial community species in different sampling groups was tested by Kruskal–Wallis H test on OTU, phyla and genera level respectively. The FDR (Falsely Discovery Rate) and Tukey’s method^16^ were used to analyze the multiple test correction for p-value > 0.95. One-way ANOVA test was used to analyze significant differences of two groups. Differences between two groups were analyzed by student’s t test. Correlation heatmap analysis of the correlation coefficient between environmental factors and selected species was determined by MeV (Multi Experiment Viewer) software (http://mev.tm4.org).

Other statistical analysis was performed using SPSS version 20.0 (SPSS Inc., Chicago, IL, USA). The figures of the microbial diversity indices and relative abundance of functional profiles were prepared using Microsoft Office 2010 (Microsoft Corporation, Redmond, WA, USA)and Adobe Illustrator CS5 (Adobe Systems Incorporated, San Jose, CA, USA) (https://www.adobe.com/cn/products/illustrator.html).

## Results

### Effects of dazomet application on watermelon *Fusarium* wilt

**Figure 1:**
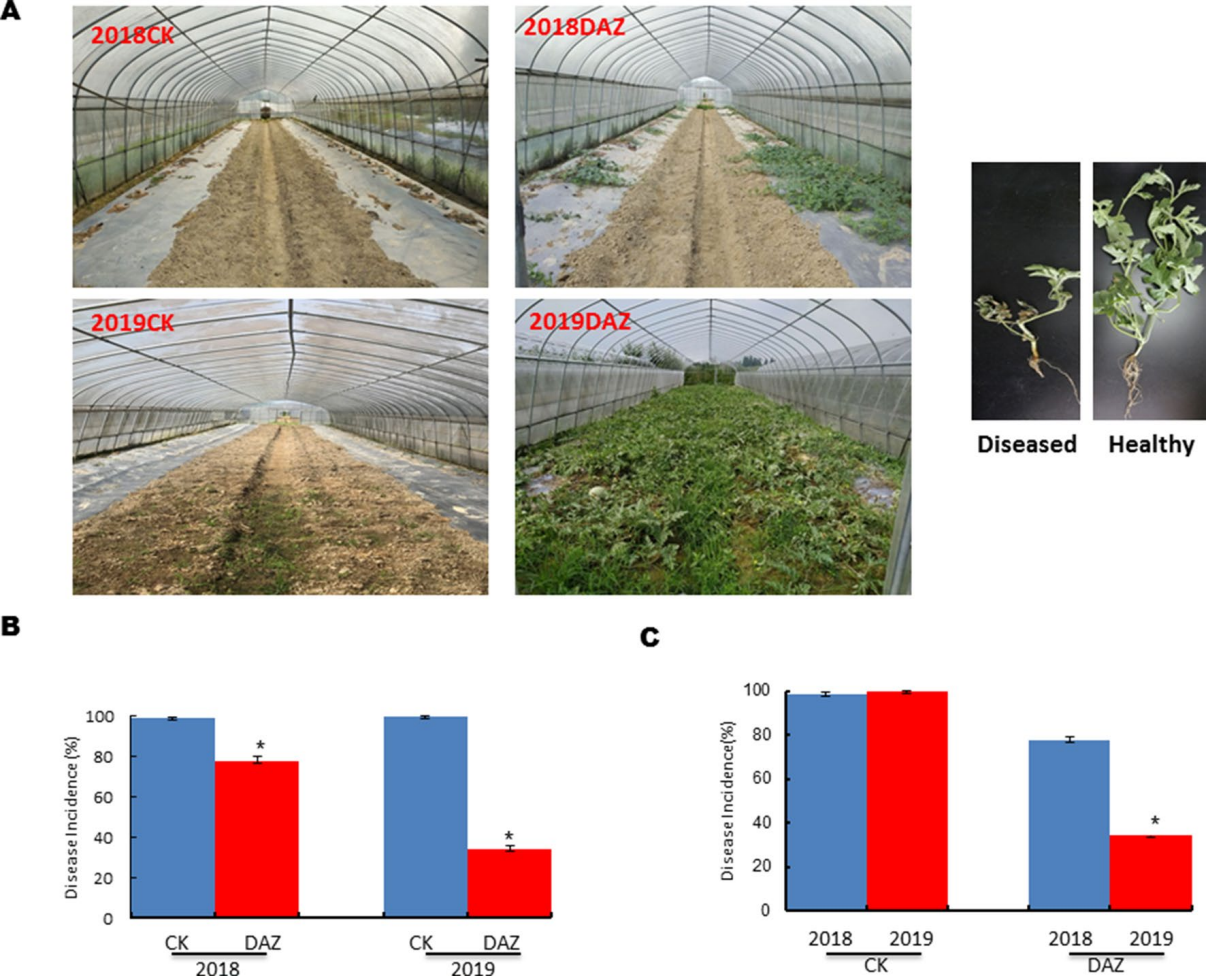
Effect of Dazomet application on Watermelon *Fusarium* wilt. (**A**) Growth of watermelon plants and plant morphology comparison in the field experiment after dazomet treatment compared with control. (**B**) Comparison of disease incidence by time effects. (**C**) Comparison of disease incidence by dazomet effects. *DAZ* dazomet treatment, *CK* control. Data are expressed as the means ± SD (n = 3). Differences between two groups were analyzed by student’s t test. *0.01 < P ≤ 0.05.

The disease incidence was significantly reduced after dazomet application compared to the control group in our filed experiment, indicating that the continuous application of dazomet could effectively suppress the occurrence of watermelon *Fusarium* wilt. As well as the plant morphological comparison between the diseased plants and healthy plants showed in Fig. 1A. Furthermore, the statistical analysis results showed that the morbidity of watermelon *Fusarium* wilt in the control group were above 98% in year 2018 and year 2019, but the disease incidences were significantly decreased after treatment with dazomet compared with control in both two years (Fig. 1B). Even more, the disease incidence in 2019 (34.17%) was much lower than that in 2018 (79.82%) after treatment with dazomet showed in Fig. 1C.

### Effects of dazomet application on soil properties of watermelon field

Results from the soil physicochemical analyses showed that there were no significant differences in the parameters, expect for EC (soil electrical conductivity) and AP (available soil phosphorous) (Supplementary Table S1). We observed that the electrical conductivity of dazomet treated soil was lower in 2019 than that of the previous year. Furthermore, we noticed that the available phosphorus content has significantly increased in the soil after dazomet application.

### Comparative analysis of soil bacterial community structure in different sampling times

#### Bacterial sequence data evaluation and OTU analysis

**Figure 2:**
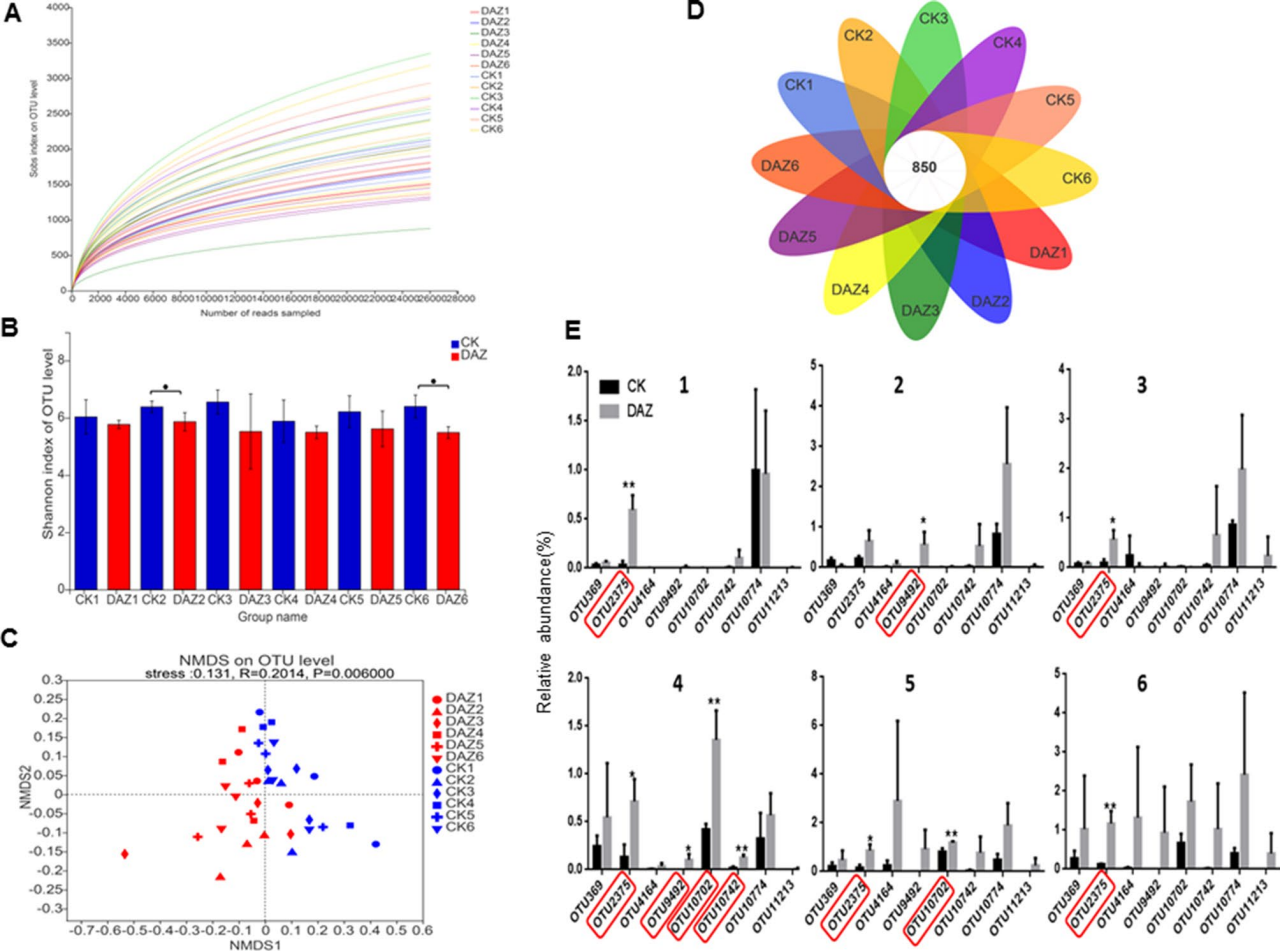
Bioinformatic Analysis of Bacterial OTUs. (**A**) The rarefaction curve of the sequencing data. Draw the curve with the extracted data volume as the abscissa and the sobs index value as the ordinate. (**B**) Significant test of OTU Shannon index between treatments for every sampling time. Data are expressed as the means ± SD (n = 3). * 0.01 < P ≤ 0.05. (**C**) NMDS to compare the bacterial community structure between treatments for every sampling time. Dots of different colors or shapes represent different groups of samples. (**D**) Venn diagram to display the number of core OTUs in all sampling groups. (**E**) Significance of core OTUs between treatments for every sampling time by student’s t test. *DAZ* dazomet treatment, *CK* control. Numbers after letters indicate different sampling times. 1 (March 6th, 2018, before dazomet treatment), 2 (April 24th, 2018, watermelon seedling stage), 3 (May 3rd, 2018, *Fusarium* wilt symptom appearance), 4 (March 6th, 2019, before dazomet treatment), 5 (April 22th, 2019, watermelon seedling stage), 6 (April 29th, 2019, *Fusarium* wilt symptom appearance).

The depth analysis of the 36 soil samples sequencing data indicated that the raw sample sequence was composed of 2,062,735 reads, clean sample sequnce with 1,491,552 reads, the average reads per sample was 41,432, and the total number of OTUs detected was 8599. The rarefaction curves of sobs index reflects the evenness of the community in the samples tends to be consistant (Fig. 2A). The student’s t-test shannon index showed that the alpha diversities of bacterial have significant differences between CK and dazomet treatment (Fig. 2B). And we found that the structures of bacterial communities have dynamics through all sampling times based on beta diversity analysis performed by NMDS (Fig. 2C). The venn diagram showed that there were 850 overlaped bacterial OTUs in all groups of samples (Fig. 2D). In addition, we found eight core OTUs had significantly dynamics based on Kruskal–Wallis H test of all sampling times. Therefore, we used the student’s t-test of shannon index to detect these eight significant differences OTUs between treatments for every sampling time (Fig. 2E). For example, the OTU2375 (p__Chloroflexi;c__Chloroflexia;o__Thermomicrobiales;f__Thermomicrobiaceae;g__Nitrolancea ) had significantly accumulated in DAZ during all the sampling times except 2. The OTU9492 (p__Actinobacteria;c__Actinobacteria;o__Micrococcales;f__Micrococcaceae;g__Arthrobacter) had increased significantly in sampling time 2 and 4. The OTU10702 (p__Proteobacteria;c__Gammaproteobacteria;o__Pseudomonadales;f__Pseudomonadaceae;g__Pseudomonas) had increased significantly in sampling time 4 and 5. And the OTU10742 (p__Firmicutes;c__Bacilli;o__Bacillales;f__Sporolactobacillaceae;g__Pullulanibacillus) had increased significantly in sampling time 4.

#### Dynamic changes of dominant bacterial at different sampling times

**Figure 3:**
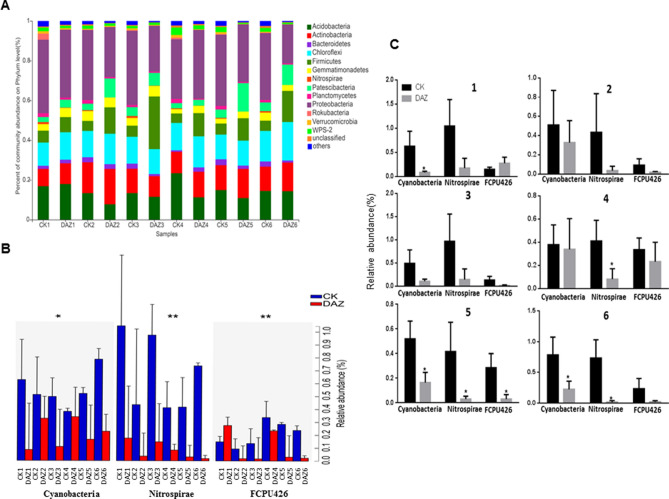
Dynamic changes of dominant bacterial community analyses on phylum level. (**A**) Community bar plot analysis of soil bacterial community abundance in different sampling times on phylum level. (**B**) Kruskal–Wallis H test was used to test the significance of the dynamic changes of bacterial community relative abundance at different sampling times on phylum level. **(C)** Student’s t-test bar plot showed significant differences of the main bacterial phyla between treatment and CK. The X-axis represents the bacterial phylum, the Y-axis represents its average relative abundance. *DAZ* dazomet treatment, *CK* control. Numbers after letters indicate different sampling times. 1 (March 6th, 2018, before dazomet treatment), 2 (April 24th, 2018, watermelon seedling stage), 3 (May 3rd, 2018, *Fusarium* wilt symptom appearance), 4 (March 6th, 2019, before dazomet treatment), 5 (April 22th, 2019, watermelon seedling stage), 6 (April 29th, 2019, *Fusarium* wilt symptom appearance). Data are expressed as the means ± SD (n = 3). * 0.01 < P ≤ 0.05, ** 0.001 < P ≤ 0.01.

**Figure 4:**
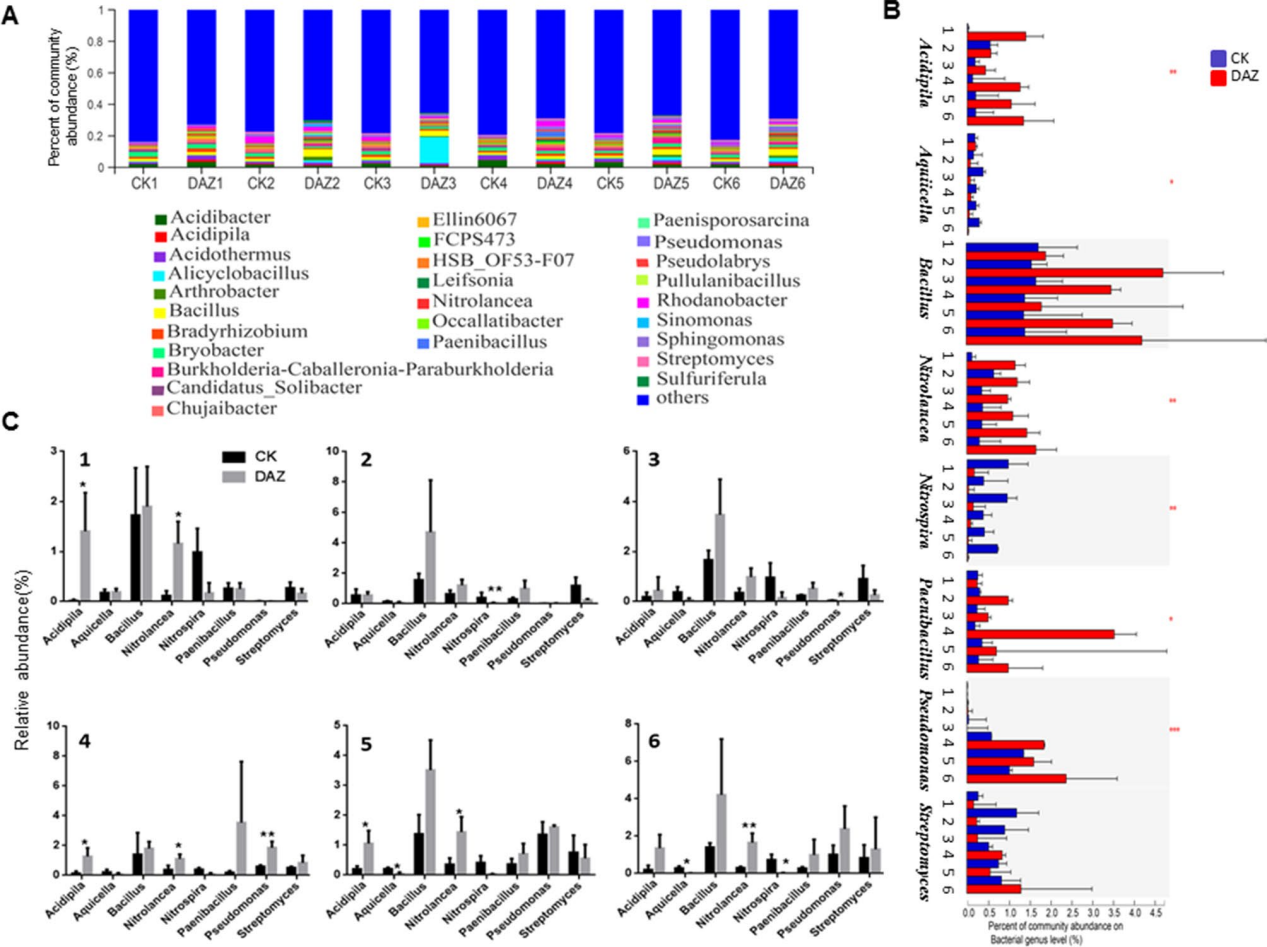
Dynamic changes of dominant bacterial community analyses on genus level. (**A**) Community bar plot analysis of soil bacterial community abundance in different sampling times on genus level. (**B**) Kruskal–Wallis H test was used to test the significance of the dynamic changes of bacterial community relative abundance at different sampling times on genus level. The X-axis represents the species, the Y-axis represents the average relative abundance of enriched communities. (**C**) Student’s t-test bar plot showed significant differences of the main bacterial genera between treatment and CK. *DAZ* dazomet treatment, *CK* control. Numbers after letters indicate different sampling times. 1 (March 6th, 2018, before dazomet treatment), 2 (April 24th, 2018, watermelon seedling stage), 3 (May 3rd, 2018, *Fusarium* wilt symptom appearance), 4 (March 6th, 2019, before dazomet treatment), 5 (April 22th, 2019, watermelon seedling stage), 6 (April 29th, 2019, *Fusarium* wilt symptom appearance). Data are expressed as the means ± SD (n = 3). * 0.01 < P ≤ 0.05, ** 0.001 < P ≤ 0.01.

To find the dynamic changes of dominant bacterial communities during all the six sampling times, we used community bar plot analysis to identify the most abundance bacterial communities both on phylum and genus levels (Figs. 3A and 4A). Furthermore, through Kruskal–Wallis H test analysis, we found that the average relative abundance of enriched phylum Cyanobacteria, Nitrospirae and FCPU426 have significantly changed after dazomet treatment (Fig. 3B). Further, we used student’s t-test to detect their significant differences between treatments for every sampling time respectively showed in Fig. 3C. Our results indicated that the Nitrospirae had decreased significantly in DAZ group in year 2019, and all these three phyla have significantly decreased in DAZ in the 5^th^ sampling time compared with CK group. Moreover, we selected 8 distributions of genera as biomarkers with significant differences based on Kruskal–Wallis H test of multiple comparisons in all sample groups to detect their dynamics. For instance, these were *Acidipila*, *Aquicella, Bacillus, Nitrolancea*, *Nitrospira*, *Paenibacillus*, *Pseudomonas* and *Streptomyces* (Fig. 4B). Notably, we identified that the community abundance of *Aquicella* and *Nitrospira* have significantly decreased after dazomet application compared to the control from year of 2019. And we spotted that the percent of *Pseudomonas* and *Nitrolancea* have significantly accumulated after dazomet treatment (Fig. 4C).

### Comparative analysis of soil fungal community structure in different sampling times

#### Fungal sequence data evaluation and OTU analysis

**Figure 5:**
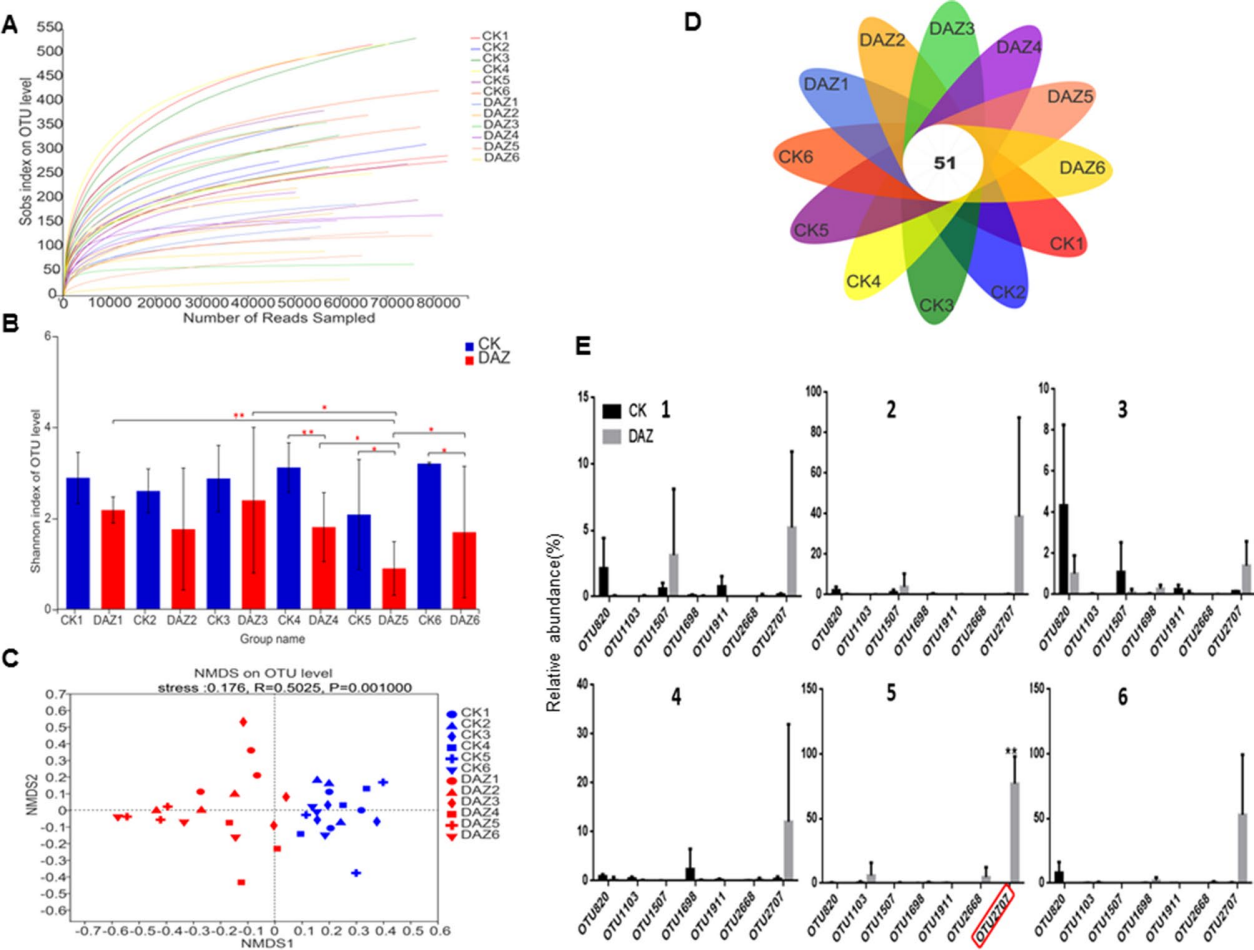
Bioinformatic analysis of fungal OTUs. (**A**) The rarefaction curve of the sequencing data. Draw the curve with the extracted data volume as the abscissa and the sobs index value as the ordinate. (**B**) Significant test of OTU Shannon index between treatments for every sampling time. Data are expressed as the means ± SD (n = 3). * 0.01 < P ≤ 0.05. (**C**) NMDS to compare the fungal community structure between treatments for every sampling time. Dots of different colors or shapes represent different groups of samples. (**D**) Venn diagram to display the number of core OTUs in all sampling groups. (**E**) Significance of core OTUs between treatments for every sampling time by student’s t test. *DAZ* dazomet treatment, *CK* control. Numbers after letters indicate different sampling times. 1 (March 6th, 2018, before dazomet treatment), 2 (April 24th, 2018, watermelon seedling stage), 3 (May 3rd, 2018, *Fusarium* wilt symptom appearance), 4 (March 6th, 2019, before dazomet treatment), 5 (April 22th, 2019, watermelon seedling stage), 6 (April 29th, 2019, *Fusarium* wilt symptom appearance).

The depth assessment analysis of the 36 soil sample sequencing data indicated that the raw sample sequence was composed of 1,318,001 reads, clean sample sequnce with 939,816 reads, the average reads of samples was 26,106 and the total number of OTUs detected was 1832. The rarefaction curves of sobs index reflects the evenness of the community in the samples tends to be consistant (Fig. 5A). The student’s t-test of shannon index showed that the alpha diversities of fungal community have significant differences between CK and DAZ treatment (Fig. 5B). And we found that the structures of fungal communities have dynamics performed by NMDS on the relative abundances of OTUs during all sampling times (Fig. 5C). The venn diagram showed that there were 50 overlaped fungal OTUs in all groups of samples (Fig. 5D). At the same time, we found eight core funagl OTUs had significantly dynamics based on Kruskal–Wallis H test of all sampling times. Then, we used the student’s t-test of shannon index to detect these seven significant differences OTUs between treatments for every sampling time. Notably, the significance test of core fungal OTUs indicated that the proportion of OTU2707(p__Ascomycota;c__Eurotiomycetes;o__Eurotiales;f__Aspergillaceae;g__Penicillium) have increased significantly in 2019 compared with their proportion in 2018 respectively (Fig. 5E).

#### Dynamic changes of dominant fungal community at different sampling times

**Figure 6:**
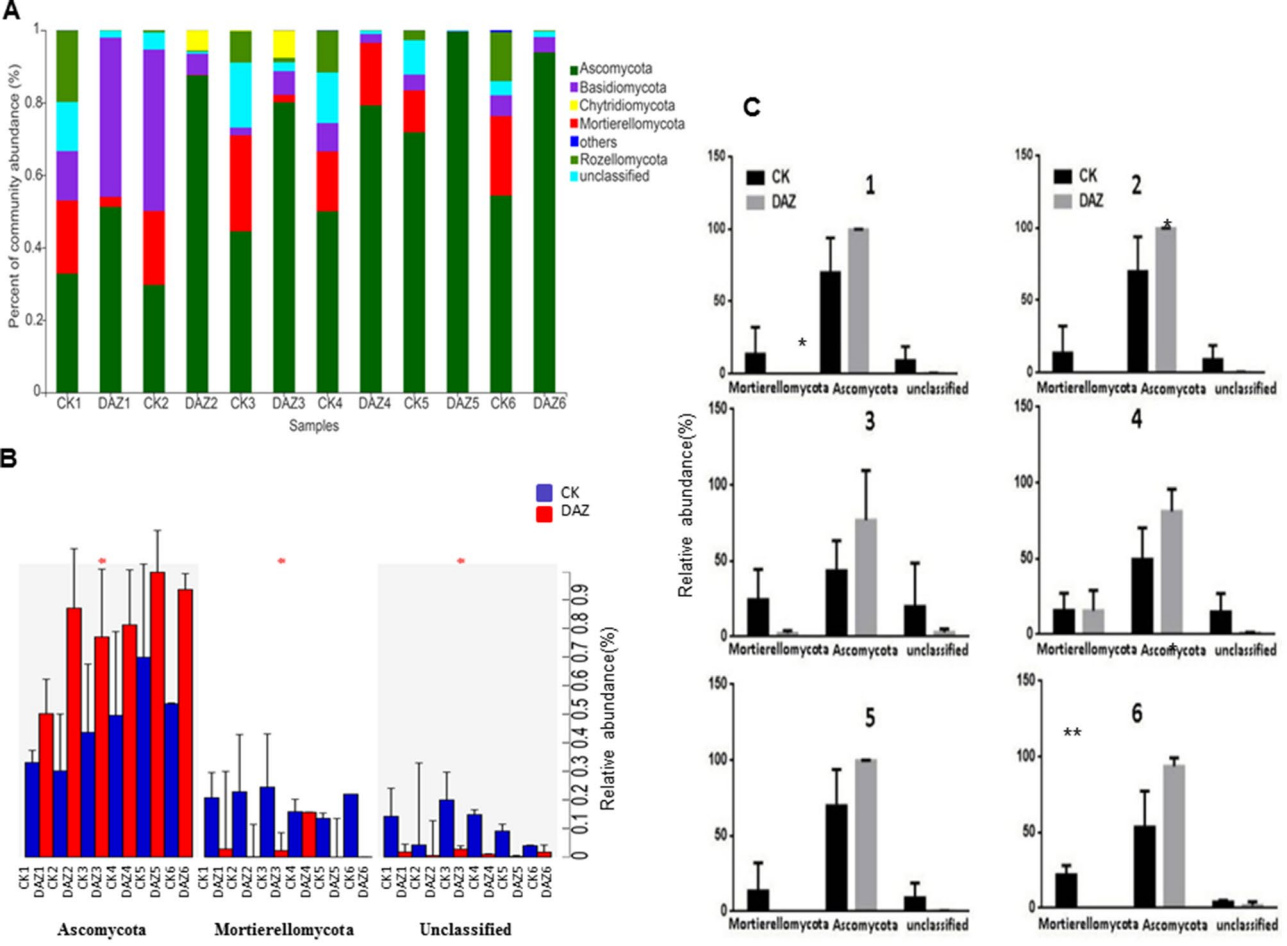
Dynamic changes of dominant fungal community analyses on phylum level. (**A**) Community bar plot analysis of soil fungal community abundance in different sampling times on phylum level. (**B**) Kruskal–Wallis H test was used to test the significance of the dynamic changes of fungal community relative abundance at different sampling times on phylum level. (**C**) Student’s t-test bar plot showed significant differences of the main fungal phyla between treatment and CK. The X-axis represents the bacterial phylum, the Y-axis represents its average relative abundance. *DAZ* dazomet treatment, *CK* control. Numbers after letters indicate different sampling times. 1 (March 6th, 2018, before dazomet treatment), 2 (April 24th, 2018, watermelon seedling stage), 3 (May 3rd, 2018, *Fusarium* wilt symptom appearance), 4 (March 6th, 2019, before dazomet treatment), 5 (April 22th, 2019, watermelon seedling stage), 6 (April 29th, 2019, *Fusarium* wilt symptom appearance). Data are expressed as the means ± SD (n = 3). * 0.01 < P ≤ 0.05, ** 0.001 < P ≤ 0.01.

**Figure 7:**
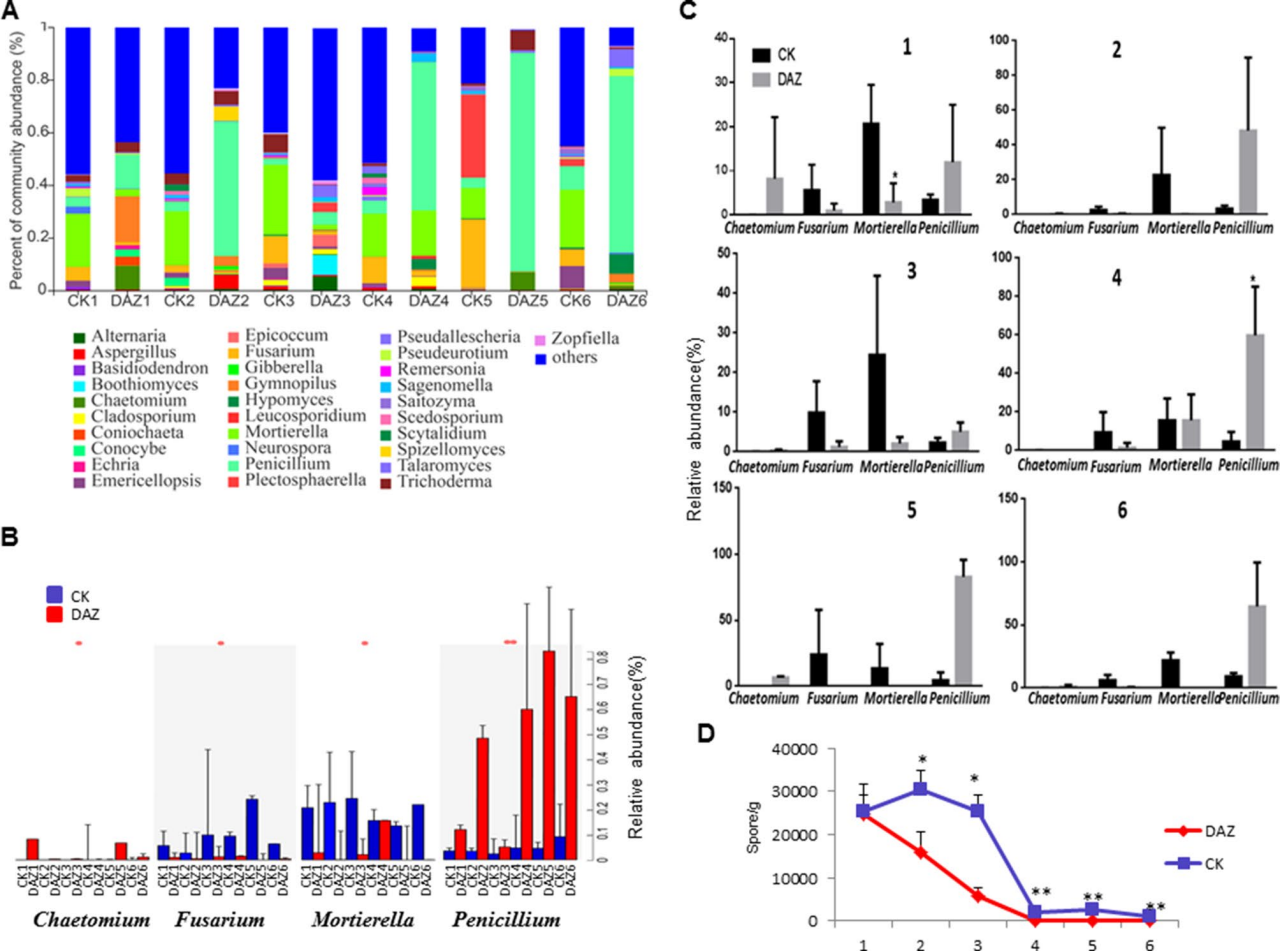
Dynamic changes of dominant fungal community analyses on genus level. (**A**) Community bar plot analysis of soil fungal community abundance in different sampling times on genus level. (**B**) Kruskal–Wallis H test was used to test the significance of the dynamic changes of fungal community relative abundance at different sampling times on genus level. The X-axis represents the species, the Y-axis represents the average relative abundance of enriched communities. (**C**) Student’s t-test bar plot showed significant differences of the main fungal genera between treatment and CK. *DAZ* dazomet treatment, *CK* control. Numbers after letters indicate different sampling times. 1 (March 6th, 2018, before dazomet treatment), 2 (April 24th, 2018, watermelon seedling stage), 3 (May 3rd, 2018, *Fusarium* wilt symptom appearance), 4 (March 6th, 2019, before dazomet treatment), 5 (April 22th, 2019, watermelon seedling stage), 6 (April 29th, 2019, *Fusarium* wilt symptom appearance). Data are expressed as the means ± SD (n = 3). * 0.01 < P ≤ 0.05, ** 0.001 < P ≤ 0.01.

In order to investigate the effect of dazomet treatment, we monitored the changes in the soil fungal communities over two years by community bar plot analysis both on phylum and genus levels respectively (Figs. 6 and 7). Based on statistical analysis showed in Fig. 6B,C, we found that the dominant phyla of Ascomycota and Mortierellomycota have significant changes after dazomet application, but their changes were on the contrary way. And at the genus level, we noticed that the percent of community abundance of *Chaetomium*, *Fusarium*, *Mortierella* and *Penicillium* have altered significantly (Fig. 7B). Notably, *Penicillium* has rapidly increased significantly after the dazomet application compared to the control group. However*,* the *Mortierella* and *Fusarium* had the decreased tendency after dazomet treatment compared to control (Fig. 7C).

### Quantitative detection FON dynamics at different sampling times by real-time PCR

For further verification the FON content in the soil at different sampling times, Q-PCR results showed that detection of pathogen FON have constantly decreased after dazomet treatment. In year of 2019, the content of FON had significantly declined in the DAZ group compared to CK (Fig. 7D).

## Discussion

In this experiment, our results showed that the disease incidence was significantly reduced after dazomet application compared to the control group. Moreover, the morbidity of watermelon *Fusarium* wilt was significantly decreased in year of 2019 than that in 2018 after dazomet application. Thus, this confirmed that dazomet application can not only effectively suppress the occurrence of *Fusarium* wilt in apple orchards^7^, cucumber^17^, ginger^6^, gooseberry^5^ but also in watermelon wilt.

Since several researches have emphasized the role of soil microbial communities in plant health and growth^9^. For example, Xin’s study used Arabidopsis mutants showed that the plant immune system was needed to maintain the normal growth of commensal bacteria^10^. Chen’s research demonstrated one of *Pseudomonas piscium* directly interfered with the molecular machinery of *Fusarium graminearum* in wheat resulting in reduced virulence and fungal growth^18^. Due to the numerous commonalities in mechanisms and principles of both types of plant–microbe interactions, scientists from both fields have started to join forces to foster an integrated view of the molecules. A nice example of this was recently demonstrated the importance of microbe-microbe interactions associated in Arabidopsis used a number of computational and microbial techniques to characterize this soil microbes and plant roots interkingdom^19^. In agree with the theory, our significance analysis of Shannon index and beta diversity analysis results indicate that the structure of not only the bacterial but also the fungal communities have altered significantly after the application of dazomet. Therefore, we can infer that the application of dazomet may active plant defense activity by shifting the soil microbial community.

Surprisingly, our comparative analysis of soil microbial community structure in different sampling times results demonstrated that the relative abundance of some dominant beneficial microbial have increased as well. For example, the bacterial of *Bacillus, Pseudomonas*, *Nitrolancea*, *Streptomyces* and the fungi genus *Penicillium*. On the contrary, the relative abundance of Fusarium genus and the pathogen FON had the decreased tendency after dazomet treatment compared to control. More specifically, numerous studies have indicated that most species of *Bacillus* promote crop yields and significantly affect the root inoculation and impact of plant growth promoting rhizobacteria on nutrient element contents^20^. Many of the polyketides and lipopeptides produced by *Bacillus* and *Paenibacillus* species have been described as antimicrobial agents that can be potentially applied as sustainable bio-organic products in medicine against human pathogens and in agriculture for controlling plant pathogens^20–22^. Some *Nitrospira* species have been reported to be involved in nitrification and nitrogen metabolism. *Nitrolancea* is a nitrite oxidizing bacteria that converts nitrite to nitrate^23^. *Pseudomonas* was recognized as one of an important beneficial microorganism to plant immunity^24–26^. For instance, Chen^18^ and colleagues demonstrated that the *Pseudomonas piscium* reduced virulence and the plant pathogenic fungus *Fusarium graminearum* growth by directly interferes. De Cal^2^ reported that *Penicillium oxalicum* effectively control of *F. oxysporum f. sp. melonis* on melon and watermelon, respectively long time ago. Taken together, the enrichment of those beneficial microbes and declined pathogen in the soil is likely the reason why the watermelon *Fusarium* wilt disease was suppressed after dazomet application.

**Figure 8:**
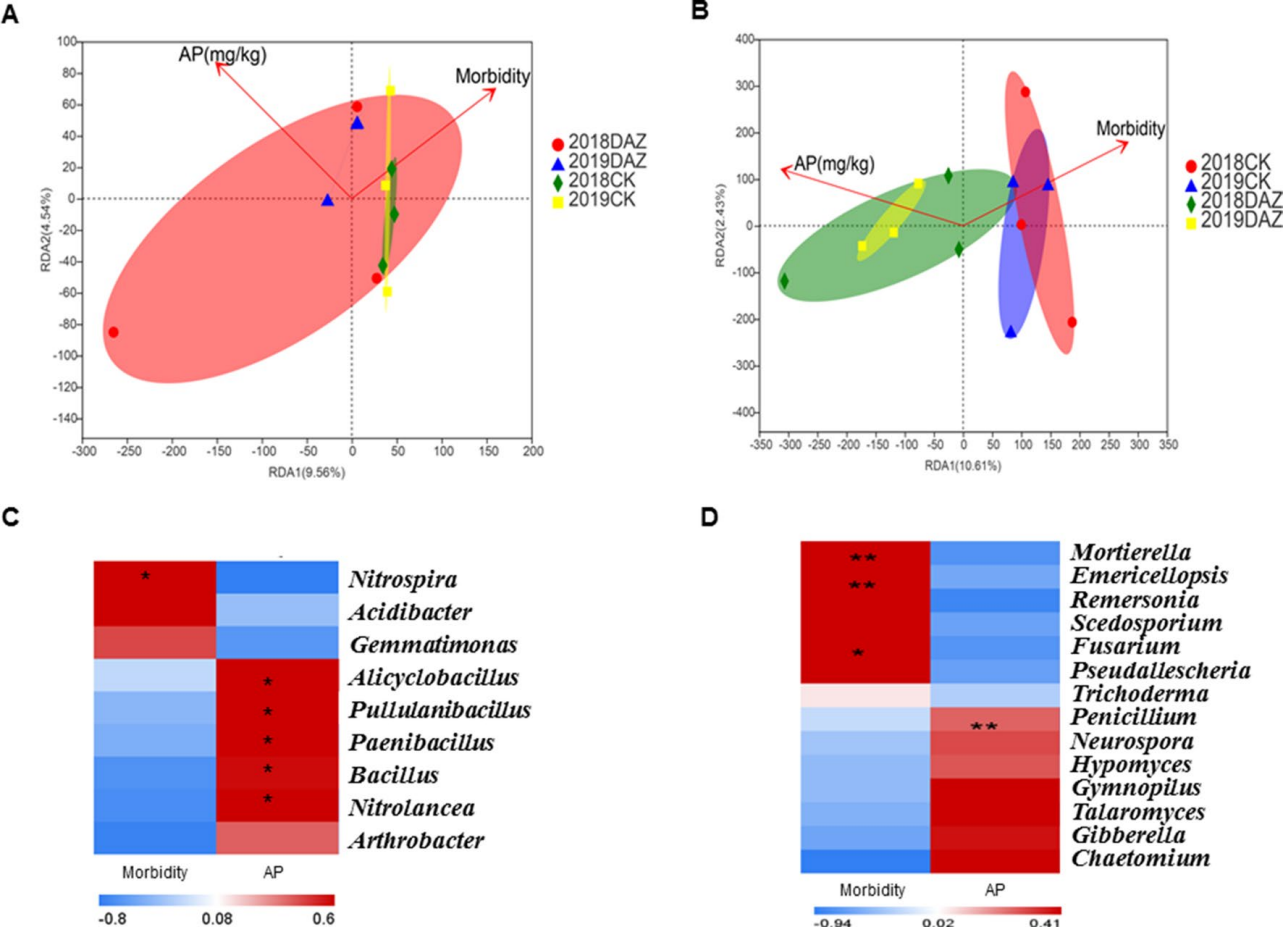
Correlation analysis of environmental factors. (**A**) RDA analysis on bacterial genus level. (**B**) RDA analysis on fungal genera level. (**C**) Spearman Correlation Heatmap of bacterial genera. (**D**) Spearman Correlation Heatmap of fungal genus. *AP* Available soil phosphorus. Red color represents highly positively correlated values; Blue color represents negatively correlated values. Means ± SD (n = 3). * 0.01 < P ≤ 0.05, ** 0.001 < P ≤ 0.01.

In addition, we noticed that the available phosphorus content has significantly increased in the soil after dazomet application. Therefore, we hypothesized that either the soil microbial community or the plant immune system might cause the AP increase. While growing evidence supports the idea that the modulation of plant immunity can reshape soil microbiota under different environmental conditions^27,28^ and plant-associated microbial communities promote plant nutrient uptake as well^25,29^. We made a correlation analysis between AP, disease incidence and soil microbial community as well. Indeed, we found that the beneficial microbes as *Paenibacillus*, *Bacillus*, *Nitrolancea* and *Penicillium* have significant positive correlation with AP but negatively related to disease incidence (Fig. 8). Moreover, the recent findings identified the root microbiota drive integration of phosphate stress and immunity^30^. They represented an important advance in showing that plant responses to phosphate deficiency are directly linked to immunity and depend on an intact root microbial. Similarity, our results enhanced the thought that plant immune system may coordinate recognizing microbial with soil phosphate during microbiome assembly. Besides, Kei Hiruma^31^ demonstrated an excellent mechanism of how the fungus *Colletotrichum tofieldiae* transfers the macronutrient phosphorus to Arabidopsis shoots. Therefore, these results may explain why dazomet management could inhibit watermelon *Fusarium* wilt disease in our study.

## Conclusion

In conclusion, this experiment demonstrates that the altered soil microbial community structure by dazomet application is critical to suppress watermelon *Fusarium* wilt. Our data also suggest two possibilities for the dynamic changing of soil microbial community structure. One reason may the enriched AP lead to stimulate plant immune system to avoid disease by assembling beneficial microorganisms therefore the new community structure has been established. Other is that dazomet application first caused the changes of AP, likely beneficial the surviving of some specific microbes that positively correlated with it which served as a layer protecting plant from pathogen attack at the same time. In addition, our results will drive investigations aimed to deploy interaction of microbiota contribute and plant immunity.

## Supplementary Information

Supplementary Information
