# Supplementary material for: Dazomet application suppressed watermelon wilt by the altered soil microbial community
Source: Sci Rep. 2020 Dec 10;10:21668. doi: 10.1038/s41598-020-78839-5 (PMC7730150; doi:10.1038/s41598-020-78839-5)
Supplement: Supplementary file 1 — Supplementary Information [file 41598_2020_78839_MOESM1_ESM.doc]

**Dazomet Application Suppressed Watermelon Wilt by the Altered Soil Microbial Community**

Feiying Zhu^1^, Jiling Xiao ^1^, Yi Zhang ^1^, Lin Wei^2^, Zhihuai Liang ^1 *^

*^1^ Hunan Agricultural Biotechnology Research Institute, Hunan Academy of Agricultural Sciences, Changsha 410125, PR China.*

*^2^* *Institute of Plant Protection, Hunan Province, Hunan Academy of Agricultural Sciences, Changsha 410125, PR China.*

Feiying Zhu, E-mail: feiyingzhu@hunaas.cn;

^*^Correspondence Zhihuai Liang, E-mail：[liangzhihuai2019@163.com](mailto:liangzhihuai2019@163.com).

**Supplementary Table S1. Comparison of soil properties**

|  | pH | N  (g/kg) | P  (g/kg) | K  (g/kg) | SOM  (g/kg) | AN  (mg/kg) | AK  (mg/kg) | AP  (mg/kg) | EC |
| --- | --- | --- | --- | --- | --- | --- | --- | --- | --- |
| 2018DAZ | 4.21±0.21a | 1.78±0.36a | 1.07±0.25a | 23.77±0.48a | 23.77±1.36a | 159.33±3.47a | 272.33±4.56a | 231.1±5.18a | 403.33±7.71a |
| 2018CK | 4.3±0.29a | 1.74±0.24a | 0.90±0.13a | 23.70±0.63a | 22.9±0.8a | 131±2.17a | 225.67±2.87a | 123.8667±5.93ab | 311.33±19.73ab |
| 2019DAZ | 4.66±0.13a | 1.80±0.27a | 1.12±0.29a | 22.37±0.34a | 23.47±1.25a | 156.67±2.31a | 225.67±6.99a | 265.0333±2.28ab | 149±2.77b |
| 2019CK | 4.59±0.4a | 1.71±0.21a | 0.9±0.08a | 22.77±0.58a | 21.5±0.95a | 133.33±2.68a | 145.33±4.28a | 136.6±2.38b | 180.3±2.89b |

DAZ: dazomet treatment; CK: control. Numbers after letters indicate different sampling times. 2018 (May 3rd, 2018, *Fusarium* wilt symptom appearance), 2019 (April 29th, 2019, *Fusarium* wilt symptom appearance). AN, available soil nitrogen; AK, available soil potassium; AP, available soil phosphorus; N, Total soil nitrogen; P, Total soil phosphorus; K, Total soil potassium; SOM, soil organic matter. Data are expressed as the means ± SD (n=3). Different lower-case letters indicate significant differences according to student’s t-test (p<0.05).
